# Chicago Academy of Medical Sciences

**Published:** 1860-03

**Authors:** 


### CHICAGO ACADEMY OF MEDICAL SCIENCES.

Academy met in Bryant's Commercial College. President Dr. Hamill in the chair. The minutes of the last meeting were read, whereupon Dr. Gore to correct his remarks as reported, by inserting the words "a post mortem examination was not deemed necessary by the Jury," instead of "was not made in consequence of objections on the part of Parents." The minutes were accepted as corrected and ordered to be recorded.

Dr. Bloodgood, from Board of Trustees, submitted estimate for Furniture, etc. for new rooms, when the whole subject was referred to the Council for final decision.

Dr. Blake's resolution, the regular subject for discussion for this evening, was next called up, and the debate upon it opened by the mover, who urged the propriety of resorting to Tracheotomy in cases of membranous croup, as offering the best chance to save the patient, and quoted some cases to sustain his position.

Dr. Bloodgood did not approve of the operation, on the ground that the remedy did not reach the disease, which extended itself below the point at which the operation was usually performed.

The discussion was continued by Drs. Bevan, Blake, and McAllister.

Dr. Powell thought the operation much more dangerous than it was usually considered, particularly from the sudden introduction of cold air into the lungs, thereby reducing the temperature of the body—as proved by an experiment performed by himself during the day upon a dog, in which the temperature was reduced three or four degrees in half an hour, which rate of loss would have proved fatal within twenty-four hours.

Dr. Gore urged the claims of Turpeth Mineral in croup, and cited a case in point recently under his charge, in which it had proved entirely successful. He opposed the use of tracheotomy, and hoped to be forgiven for ever having advised it.

Dr. Davis approved the use of the Turpeth Mineral, which had proved very successful in his hands during a period of nine years. He was in the habit of urging its claims in his lectures to his class, and he relied mainly upon it in the treatment of this disease.

Dr. Hay gave notice of his intention to move at the next meeting certain alterations in the By-Laws.

Dr. Bevan (Treasurer) read a report of the financial condition of the Academy to date, when the accounts had passed into his hands.

The Secretary reported that in compliance with the instructions of the Academy issued at their last meeting, he had filed with the Clerk of the County Court of Cook County, a certificate of the organization of the Chicago Academy of Medical Sciences, as required by law to constitute this Academy a corporate body. A copy of this certificate was submitted and ordered to be filed with the archives of the Academy.

On motion of Dr. Jones, the subject for discussion at the next meeting was decided to be "Whether the Ferruginous Tonics are useful or hurtful in cases of Chlorosis occurring in Strumous subjects."

The President called upon the council to meet at Dr. Bloodgood's Office, on Friday Evening next.

Upon motion, the Academy adjourned.

